# Coupling of Fused Deposition Modeling and Inkjet Printing to Produce Drug Loaded 3D Printed Tablets

**DOI:** 10.3390/pharmaceutics14010159

**Published:** 2022-01-10

**Authors:** Laura Andrade Junqueira, Atabak Ghanizadeh Tabriz, Francisco José Raposo, Luana Rocha Carobini, Urias Pardócimo Vaz, Marcos Antônio Fernandes Brandão, Dennis Douroumis, Nádia Rezende Barbosa Raposo

**Affiliations:** 1 Núcleo de Pesquisa e Inovação em Ciências da Saúde (NUPICS), Universidade Federal de Juiz de Fora, Juiz de Fora 36036-900, Brazil; laura.deandrade@hotmail.com (L.A.J.); ffox3000@gmail.com (F.J.R.); luanacarobini@gmail.com (L.R.C.); pardocimo@gmail.com (U.P.V.); marcosbrand2012@gmail.com (M.A.F.B.); 2 Faculty of Engineering and Science, School of Science, University of Greenwich, Chatham Maritime, Chatham ME4 4TB, UK; ata_ghanizadeh@hotmail.com (A.G.T.); d.douroumis@greenwich.ac.uk (D.D.)

**Keywords:** three-dimensional printing, fused deposition modeling, inkjet printing, polyvinyl alcohol, minoxidil, dissolution

## Abstract

In the current study, we have coupled Fused Deposition Modelling (FDM) for the fabrication of plain polyvinyl alcohol (PVA) tablets followed by dispensing of minoxidil ethanolic solutions using inkjet printing. The use of a drop-on-solid printing approach facilitates an accurate and reproducible process while it controls the deposition of the drug amounts. For the purpose of the study, the effect of the solvent was investigated and minoxidil ink solutions of ethanol 70% *v*/*v* (P70) or absolute ethanol (P100) were applied on the plain PVA tablets. Physicochemical characterization showed that solvent miscibility with the polymer substrate plays a key role and can lead to the formation of drug crystals on the surface or drug absorption in the polymer matrix. The produced minoxidil tablets showed sustained release profiles or initial bursts strongly affected by the solvent grade used for dispensing the required dose on drug loaded 3D printed tablets. This paradigm demonstrates that the coupling of FDM and inkjet printing technologies could be used for rapid development of personalized dosage forms.

## 1. Introduction

Three-dimensional printing (3DP) is a digitally controlled process used for fabrication of 3D objects, which are produced in a layer-by-layer manner. It is used in industries such as automobiles, robotics and, aerospace. During the past decade, the technology received a great deal of attention within the medical field, in categories such as surgical planning [1], prostheses [2], medical education/training [3], organ/tissue bioprinting [4,5], and pharmaceuticals [6,7,8]. Since the Food and Drugs Administration’s (FDA) approval of the Aprecia Pharmaceutical’s Spritam^®^ (levetiracetam), the first available 3D printed tablet on the market, the interest of the pharmaceutical industry in printable oral dosage forms has grown significantly [9]. The possibility of personalization, at a reduced cost, is one of the advantages of the 3DP in the pharmaceutical field. Therefore, medicines can be produced in specified doses and geometries for each individual, according to their characteristics, needs, and preferences, to enhance the patient’s compliance, particularly for the elderly and pediatrics [10,11,12]. Additionally, 3DP allows for an increase in the complexity of the medicine’s geometry, due to the digital control over the structure of the object. Drug release can be affected by the medicine structure, which can create new options for drug delivery [13,14,15].

In the course of the 3DP history, different techniques have been developed, such as stereolithography (SLA), selective laser sintering (SLS), and FDM [10]. The materials deposited in the 3DP process can be powders, plastics, ceramics, metals, liquids, or living cells, making the process highly versatile [11]. Despite the variety of techniques and materials, most 3D printing processes follow the same basic procedure to fabricate an object. Briefly, the steps are: (i) development of the 3D object design with computer-aided design software; (ii) conversion of the 3D design into a file format recognizable by the printer, such as stereolithography file (STL); (iii) the printer software performs the slicing of the object in several layers; and (iv) fabrication of the object in a layer-by-layer manner, according to the specific printing method [10,14].

Amongst the 3DP technologies, FDM is the most commonly used. Based on an extrusion system, it uses a thermoplastic polymeric filament to build 3D objects. Briefly, the filament passes through a heated nozzle, which causes it to melt or turn semi-solid, then the material is deposited on the platform, layer by layer, forming the object [16]. In the past, FDM was applied for non-pharmaceutical purposes. Nowadays, there are several pharmaceutical grade polymers used in FDM technology such as ethyl cellulose, hydroxypropyl cellulose, hydroxypropyl methyl cellulose, hydroxypropyl methyl cellulose acetate succinate, ethylated acrylate copolymer, polyethylene glycol, polyethylene oxide, polylactic acid, PVA, and polyvinyl pyrrolidone [17,18,19,20,21].

PVA is a water-soluble polymer frequently used as a support material in the 3DP of complex objects in FDM technology [22]. Once printing is complete, the support structures are easily dissolved in water, which can then be disposed of conveniently through normal waste water systems due to their biodegradable nature. Moreover, PVA is already used as a safe excipient in the pharmaceutical industry [23]. It possesses excellent mechanical and thermal properties, which enables filament production [24]. PVA has been widely used in the 3DP of medicines [25,26,27], due to its biocompatibility, biodegradability, and more importantly, being approved by FDA [28,29]. Besides water, PVA is slightly soluble in ethanol and insoluble in many organic solvents [28].

In order to produce drug-loaded tablets using FDM, it is necessary to incorporate the drug into the filament which can be achieved by either using hot melt extrusion (HME) or by tablet impregnation as an alternative. HME produces a drug loaded filaments by applying thermal processing of the drug/polymer blends, while the impregnation uses a concentrate drug solution, and the drug is incorporated into the filament by passive diffusion. Impregnation is a simple method that does not require any additional equipment. However, it presents some disadvantages such as the need for highly concentrated drug solutions, limited dosage targeting, time-consuming, and the possibility of drug degradation [24,30,31]. Previously, we developed an alternative method that uses 3D printed tablets as scaffolds, and the drug solution is applied on the tablet surface with a micropipette [32]. This method is rapid and allows the use of the deposition of drug amounts, avoiding any wastage while it can be used for thermolabile drugs. However, the process of the drug solution application is manual, which is subject to inevitable operational human errors [33].

In order to overcome this drawback, it is crucial to use an automated, accurate and reproducible procedure. Inkjet printing is a process that digitally controls and deposits small liquid droplets on a substrate [34]. It is a high-speed fully automated process that provides accurate and precise control over the droplet size, which in turn minimizes material losses. Moreover, allows the design of various deposition patterns and facilitates complex drug release profiles [35]. Thus, the objective of this work was to couple FDM and inkjet printing to produce minoxidil tablets. FDM was used to produce the plain tablets, using PVA as the filament, and inkjet printing was used to deposit minoxidil ethanolic solution on the tablet surface. Moreover, we evaluated the influence of ethanol concentration (ethanol 70% *v*/*v* and absolute) on tablet dissolution. The coupling of FDM, for the printing of plain tablets, and inkjet printing for the dispensing of drug solutions on the tablet substrate could be effectively used for the development of personalized dosage forms. Thus, this work presents a platform that can be used for point of care applications to produce drug loaded tablets for a wide range of drugs at the required dose, enabling the dose adjustment according to the patient’s needs and also the control of the release profile.

## 2. Materials and Methods

### 2.1. Materials

Minoxidil sulfate (Fagron, São Paulo, Brazil) was used as the model drug. A commercially available PVA filament (Shenzhen Esun Industrial Co., Ltd., Shenzhen, China) was used to produce the plain tablets. Organic solvents, buffering reagents and all other substances used were of analytical grade and obtained from Sigma Aldrich (St. Louis, MO, USA).

### 2.2. Thermogravimetric Analysis (TGA)

In order to investigate the thermal stability of PVA and minoxidil, a TGA instrument was utilized (Discovery 5500, Thermal Instruments, Trevose, PA, USA). Approximately 2–2.5 mg of the bulk samples were weighed and located in a standard aluminum TGA pan. The samples were then examined from room temperature to 400 °C at a heating rate of 10 °C/min. The generated data were analyzed via TA Universal Analysis software (Universal Analysis 2000, version 4.5A, TA instruments, Trevose, PA, USA).

### 2.3. Differential Scanning Calorimetry (DSC)

To investigate the thermal behavior of the bulk materials DSC (Mettler-Toledo 823e, Greifensee, Switzerland) was utilized. Approximately 2–2.5 mg of the samples were weighed and placed into a standard 40 µL aluminum DSC pan and crimped promptly. The samples were then heated from 0 °C to 300 °C at a heating rate of 10 °C/min. The generated DSC thermograms were analyzed using the STARe Excellence Thermal Analysis software (Version 10.00, Mettler Toledo, Greifensee, Switzerland).

### 2.4. X-ray Powder Diffraction (XRPD)

X-ray powder diffraction was utilized to investigate the physical state of the minoxidil and PVA filament. The XRPD data were collected using a D8 Advance X-ray Diffractometer (Bruker, Karlsruhe, Germany) equipped with a LynxEye silicon strip position sensitive detector and parallel beam optics. The diffractometer was operated with a transmission geometry using Cu Kα radiation at 40 kV and 40 mA. The instrument was computer-controlled using XRD commander software (Version 2.6.1, Bruker AXS, Karlsruhe, Germany) and the data were analyzed using the EVA software (version 5.2.0.3, Bruker AXS, Karlsruhe, Germany). The minoxidil was ground using a mortar and pestle and placed into an aluminum sample holder. Diffraction data was collected between 2–60° 2θ with a step size of 0.02° and a counting time of 0.3 s per step. The PVA was placed between foils of 2.5 µm thick mylar for measurement. Data were collected between 5–60° 2θ with a step size of 0.04° and a counting time of 0.2 s per step.

### 2.5. Design and 3D Printing of the Plain PVA Tablets

Plain tablets (disk geometry) were designed (diameter: 12 mm; height: 3 mm; depth: 2 mm) using a 3D CAD software [SolidWorks^®^ 2015 (Dassault Systèmes SolidWorks Corporation, Waltham, MA, USA)]. The tablet size was carefully designed for oral intake according to the Food and Drugs Administration (FDA), which recommends that the largest dimension of a tablet or capsule should not exceed 22 mm [36]. The design was then exported as a STL file into the slicing software (Flashprint, FlashForge, Jinhua City, Zhejiang Province, China). A Dreamer NX (FlashForge; Oderço Distribuidora de Eletrônicos Ltd.a, Maringá, Brazil) printer was used to fabricate the plain PVA tablets, with the following printing parameters: extrusion temperature (205 °C), build plate temperature (50 °C), infill percentage (100%), infill pattern (line), and printing speed (50 mm/s), with the aid of a raft. A commercially available PVA filament was used to produce the plain tables.

### 2.6. Evaluation of Printing Reproducibility

To assess the printing reproducibility of the structures, 20 plain tablets were printed and their dimensions were measured across the diameter, height, and depth with the aid of a digital caliper (Mitutoyo Sul América Ltd.a., Suzano, Brazil). Results were expressed as mean, standard deviation (SD), and relative standard deviation (RSD).

### 2.7. Deposition of Minoxidil Solution by Inkjet Printing

The plain 3D printed PVA tablets were used to produce the minoxidil loaded tablets (1 mg per tablet). An ethanolic solution of minoxidil was deposited on top of the plain tablets within the cavity via inkjet printing. A Nanoplotter II (GeSim GmbH, Dresden, Germany) fitted with a PicPip 300 dispenser was used, where the drug (1% *w*/*v*) was dissolved in two different EtOH grades (70% *v*/*v* and absolute). The inkjet printer comprises of the wash station, dispenser, and dispense control system. An accurate flow sensor controls the amount of aspiration, while a stroboscope ensures that the dispenser ejects the droplets. Before and after sample dispensing, stroboscopic image capture allows for real-time observation of the nozzle performance. Two types of minoxidil tablets were produced by applying 100 μL of a minoxidil solution (1% *w*/*v*) with ethanol 70% *v*/*v*–P70–and a second batch with minoxidil solution (1% *w*/*v*) in absolute ethanol–P100.

### 2.8. Dissolution Study and Ultraviolet-Visible (UV–VIS) Spectroscopy Analysis

The dissolution study was performed according to USP 42, with modifications. A USP apparatus 2 (paddle) from Hanson Research, SRII-6, USA was used for dissolution studies. The minoxidil tablets (*n* = 6 for each type) were immersed in 900 mL of phosphate buffer pH 7.2, at 37 °C, and stirring speed set to 75 rpm. Then, aliquots of 10 mL were collected at 5, 15, 30, 60, 90, and 120 min and analyzed by UV–VIS spectroscopy. The amount of minoxidil into the samples was quantified using a microplate reader (Multiskan GO, Thermo Fisher Scientific, Waltham, MA, USA) at the wavelength of maximum absorption (λmax = 227 nm). For the dissolution studies, a calibration curve was plotted using five different minoxidil concentrations (0.707, 0.859, 1.010, 1.162, and 1.313 μg mL^−1^). All samples were filtrated in a 0.22 μm filter (Kasvi, São José dos Pinhais, Brazil) prior the analysis.

### 2.9. Determination of Minoxidil

The drug content and uniformity of the minoxidil tablets were carried out. For drug content determination, the tablets (*n* = 5 for each type) were placed in pH 7.2 phosphate buffer solution and shaken with magnetic bars for two hours. The solutions were diluted (final concentration = 1 μg mL^−1^) and quantified. For the evaluation of the uniformity, the tablets (*n* = 10 for each type) were individually evaluated, using the same method of content determination. The minoxidil was determined by UV–VIS spectroscopy analysis and the quantification was performed by direct comparison with the standard at the same concentration.

### 2.10. Weight Variation

The minoxidil tablets (*n* = 20) were weighed on the analytical scale (Y220, Shimadzu, Kyoto, Japan). The average weight and percentage of weight variation was calculated.

### 2.11. Friability

The impregnation method could lead to the loss of minoxidil during handling/packaging since it could be on the surface of the tablet. To evaluate this, the friability test was performed. The minoxidil tablets (*n* = 20 for each type) were weighed and placed carefully in an Ethik 300 friability apparatus (Ethik Technology, São Paulo, Brazil). The tester rotated with 25 rpm for 4 min. The tablets were weighed again and the relative weight loss was calculated following Equation (1):

F = ((W_i_ − W_f_)/W_i_) × 100 (1)

where F is the friability, W_i_ is the initial weight of the tablets, and W_f_ is the tablet weight after the tests.

### 2.12. Scanning Electron Microscopy (SEM)

Scanning electron microscopy (Hitachi SU8030, Tokyo, Japan) was used to evaluate the print quality of the plain PVA tablets. Moreover, the analysis was performed before and after the impregnation, to assess changes in the tablet surface. The 3D printed sample was attached to an aluminum stub using a conductive carbon adhesive tape (Agar Scientific, Stansted, UK). The SEM images of the examined samples were captured with an electron beam accelerating voltage of 1 KV with 30×, 40× and 2000× magnifications.

## 3. Results and Discussion

### 3.1. Thermal Characterization

**Figure 1:**
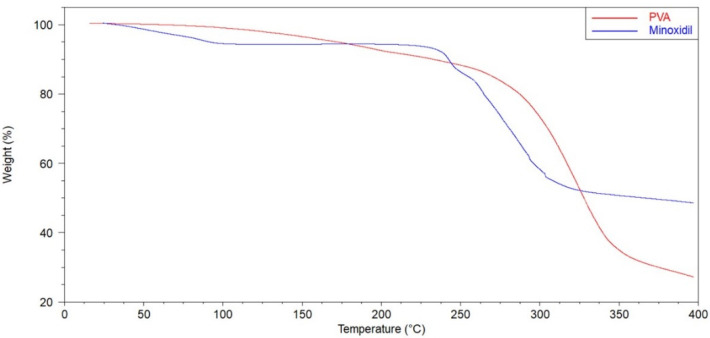
TGA thermograms of PVA and Minoxidil.

The thermal stability of the PVA and minoxidil was evaluated. The TGA data (Figure 1) showed that the PVA undergo an initial decomposition at the temperature range of 90–190 °C, due to the volatilization of free water and bound water [37]. Then, occurs a sharp degradation at the temperature range of 250–375 °C, which represents the more significant weight loss due to degradation of side group (–OH) [38]. Thus, in the printing temperature (205 °C), PVA is thermally stable. Minoxidil also has an initial water loss of approximately 5.6%, remaining thermally stable up to 237 °C. The drug has not been submitted to high temperatures since it was deposited in the plain tablets by inkjet printer.

**Figure 2:**
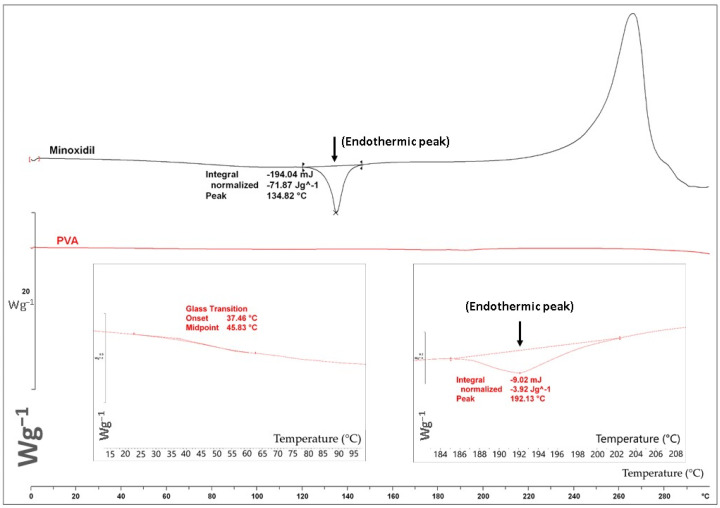
DSC thermograms of minoxidil and PVA.

The DSC thermograms of PVA and minoxidil are presented in Figure 2. PVA presented a major melting endothermic peak at 192.13 °C, and a step change at 45.83 °C, which can be attributed to the glass transition temperature (Tg). The thermal profile is representative of the semicrystalline nature of PVA due to the presence of amorphous and crystalline regions [39]. Minoxidil exhibited a melting endotherm at 134.82 °C, which is in accordance with the literature (Tm: 143.5 °C) [40]. Moreover, it presented an exothermic crystallization peak at 262.45 °C.

### 3.2. X-ray Powder Diffraction (XRPD)

**Figure 3:**
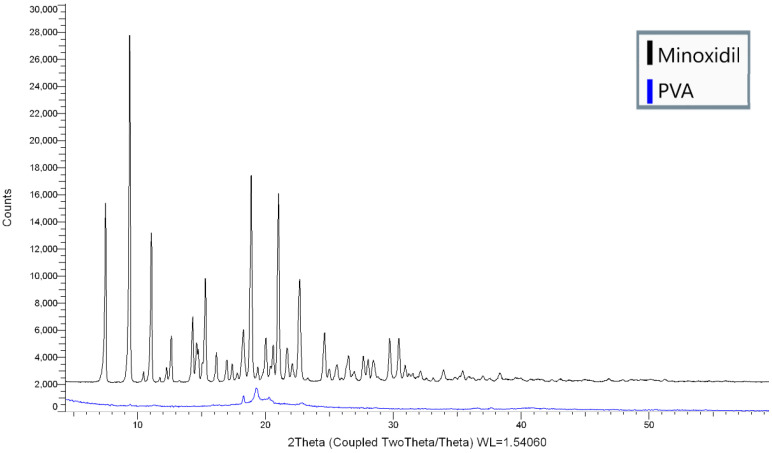
XRD patterns of minoxidil and PVA.

X-ray powder diffraction patterns of PVA and minoxidil were collected and are shown in Figure 3. The diffractogram of PVA suggests its semicrystalline nature, with a characteristic broad peak at 2θ = 19.89° [41,42]. This result is in accordance with DSC analysis since both demonstrate the partial crystallinity of PVA. The diffractogram of crystalline minoxidil presented sharp intensity peaks at 7.5°, 9.4°, 11.08°, 15.0°, 18.9°, 21.0°, and 22.7/2θ°. As minoxidil did not undergo any processing (HME, 3DP), it remained in crystalline form within the tablet.

### 3.3. 3D Printing of the Plain PVA Tablets

Several 3DP techniques have been reported for the printing of oral solid formulations, such as FDM, SLS, SLA, and inkjet 3DP. FDM is the most frequently used, were around 76% of the studies for oral solid dosage forms refer to this technology [43]. In this work, FDM was selected because it is a fast, effective and easy to use technology that enables the fabrication of tablets with complex geometries. Moreover, it allows the production of oral solid dosages with targeted release profiles (immediate or extended) without additional coating, through the adjustment of the polymer formulation and/or the tablet shape and structure [44].

**Figure 4:**
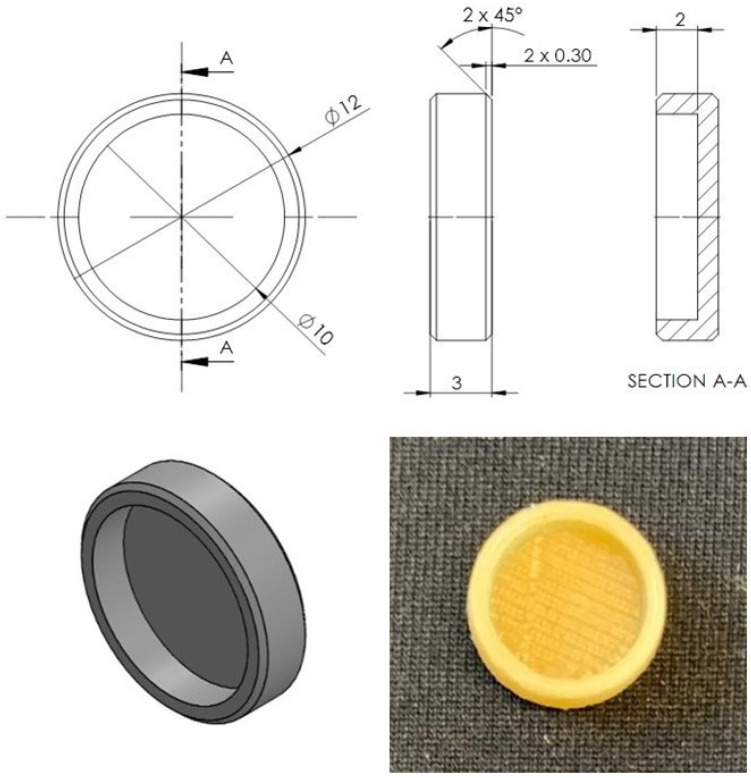
Design and picture of the plain tablet produced with the PVA filament.

The design of the plain tablet produced using the FDM and the PVA filament can be seen in Figure 4. The tablets were designed with a cavity on the top in order to accommodate the drug solution. The results of the printing reproducibility are shown in Table 1 where the plain tablet dimeter, height, and depth were measured in order to evaluate the printing reproducibility. As it can be seen, FDM demonstrated low variability (RSD ˂ 1), which suggests excellent printing reproducibility. Nevertheless, the dimensions of plain tablets are in accordance with the designed in the CAD file.

### 3.4. Friability

The friability was used to evaluate the tablet mechanic resistance to abrasion, capping, and chipping that can happen during manufacturing, packaging, and shipping. The results (P100 = 0.30%; P70 = 0.37%) demonstrated that the minoxidil tablets were resistant and met USP specifications (weight loss ≤ 1%) [45]. Moreover, other researchers have also reported friability values within the USP specifications, for tablets produced by FDM or other 3DP technologies. Cerda et al. found friability below 0.1% [27] while Sharma et al. observed no loss in friability for 3D printed tablets produced by FDM using PVA in both cases [46]. Khaled et al. demonstrated friability in a range of 0.59 to 0.65 for paracetamol tablets with different geometries [47]. However, in our case, the observed friability was attributed to the drug loss from the tablet surface due to the weak adherence of the drug. This suggests that drug adherence should be improved in future studies, for example, through the addition of polymer in the drug solution.

### 3.5. Deposition of Minoxidil Solution by Inkjet Printing

**Figure 5:**
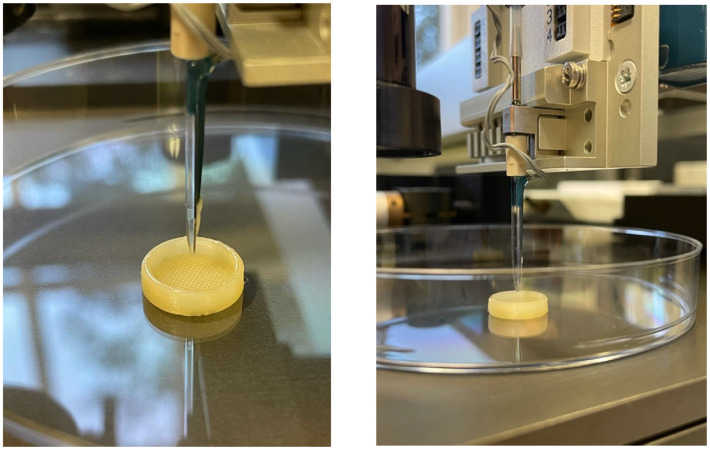
Deposition of minoxidil ethanolic solution by inkjet printing on plain PVA tablets made by FDM.

In the present work, inkjet printing was used to deposit the drug solution, by ejecting small droplets, in the plain tablets surface (Figure 5). Based on previous work the pulse duration was adjusted at 60 μs and the voltage at 100 V, while the designed algorithm allowed jetting of one droplet/50 μm [48,49]. EtOH was a good solvent for inkjet printing due to the high boiling point (78.37 °C), which prevented clogging of the nozzle. In addition, the system was programmed to wash the tip regularly to avoid any drug accumulation due to fast solvent evaporation. The inkjet pattern was particularly designed to apply several dispensing rounds for the application of the drug solution. However, due to the very small droplet size, the solvent was evaporated before the return of the nozzle back in the starting spot.

Herein, minoxidil at low doses was used as the model, aiming for alopecia treatment. The drug was initially used in the 1970s as an antihypertensive, but it was observed to cause a common adverse event, hypertrichosis. The observation of this side effect led to the development of a topical formulations for promoting hair growth, and nowadays is the main treatment for androgenetic alopecia and hair loss conditions as an off-label treatment [50]. Although topical minoxidil is an effective treatment, it presents some drawbacks, such as the necessity to apply the medication twice a day while it causes undesirable hair texture, and scalp irritation [51]. Hence, the use of oral minoxidil for the treatment of alopecia has received increasing attention [52,53,54]. However, a low dose of minoxidil is needed since potential adverse effects could occur with doses between 10 and 40 mg daily [51]. Literature suggests women require lower doses, from 0.25 to 2.5 mg daily, while men require higher doses for maximal efficacy, from 1.25 to 5 mg a day [55]. The combination of 3D printed dosage forms with inkjet printing can personalize low-dose minoxidil tablets with the required dosing regimens, at a reduced cost, taking into account the differences in dosages for men and women. Furthermore, drug amounts can be applied with high accuracy and reproducibility, while dose dispensing is rapid.

Indeed, inkjet printing on P70 tablet demonstrated minoxidil content of 92.33% and uniformity of 103.60% (SD = 2.72; RSD = 2.63), while for P100 tablets the minoxidil content was 99.04% and uniformity of 103.30% (SD = 4.84; RSD = 4.69). The results of the weight variation are shown in Table 2 where all minoxidil tablets showed acceptable weight variation in agreement with the USP specification (±7.5%) for uncoated tablets and average weight 130–324 mg [45,47].

### 3.6. Dissolution Study

**Figure 6:**
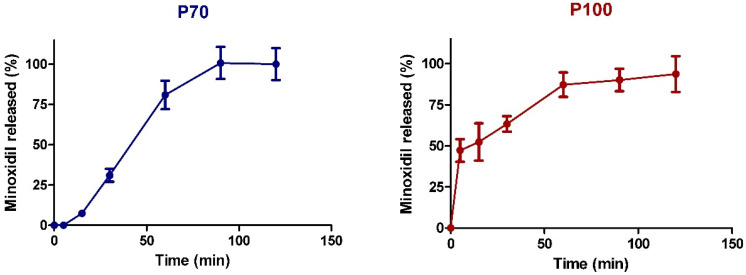
Minoxidil release profiles from P70 and P100 tablets in phosphate buffer pH 7.2.

The main objective for using different ethanol grades, was to investigate their effect on the release profiles of minoxidil from the drug loaded 3D printed tablets. The results of the dissolution study are shown in Figure 6. As PVA is a water-soluble polymer, both tablets (P70 and P100) gradually dissolved in the phosphate buffer throughout the dissolution studies. The drug loaded 3D printed tablets were completely dissolved within two hours. The minoxidil powder (alone) was used as the control, and presented 92.38% of dissolution, within 5 min.

As shown in Figure 6 minoxidil presented different dissolution profiles depending on the applied EtOH ink grade. The P70 tablets showed a sustained release over a period of 90 min where 100% of the drug was detected. On the other hand, the P100 tablets demonstrated a two-phase release profile, in which an initial burst release occurred, where around 50% of the drug was detected within the first 5 min, followed by sustained release for 120 min. This type of release profile could be of use in cases where a relatively large dose of the drug is initially needed for rapid onset of action, followed by sustained release in order to maintain the required drug levels [56].

The difference in the release profiles of the P100 and P70 tablets is attributed to the difference of the PVA solubility in water and ethanol. The P100 tablets were produced using absolute ethanol where PVA is slightly soluble in this solvent.

Thus, it is assumed that a portion of the minoxidil remained on the surface of the tablet and released quickly in the first few minutes. However, the rest of the drug penetrated the polymer and was released at the same rate as the tablet was dissolved in the dissolution media. In contrast, the P70 tables were produced using 70% (*v*/*v*) ethanol where PVA is more soluble. The higher water content of P70 allowed PVA solubilization and minoxidil absorption in the tablet. Thus, the drug was released at a constant rate, by following a similar rate to the tablet disintegration. It is clear that the PVA characteristics were essential for the obtained release of minoxidil. In previous work, a PLA filament was used, and the 3D printed tablets were impregnated in the drug solution. However, the drug was released within 5 min, as PLA is not soluble in ethanol, thus the drug remained on the tablet surface [32]. Tagami et al. (2019) [57] evaluated the influence of different solvents on the 3D production of curcumin tablets and demonstrated the effect of the solubilization of the printed polymer in the selected solvent.

### 3.7. Scanning Electron Microscopy (SEM)

**Figure 7:**
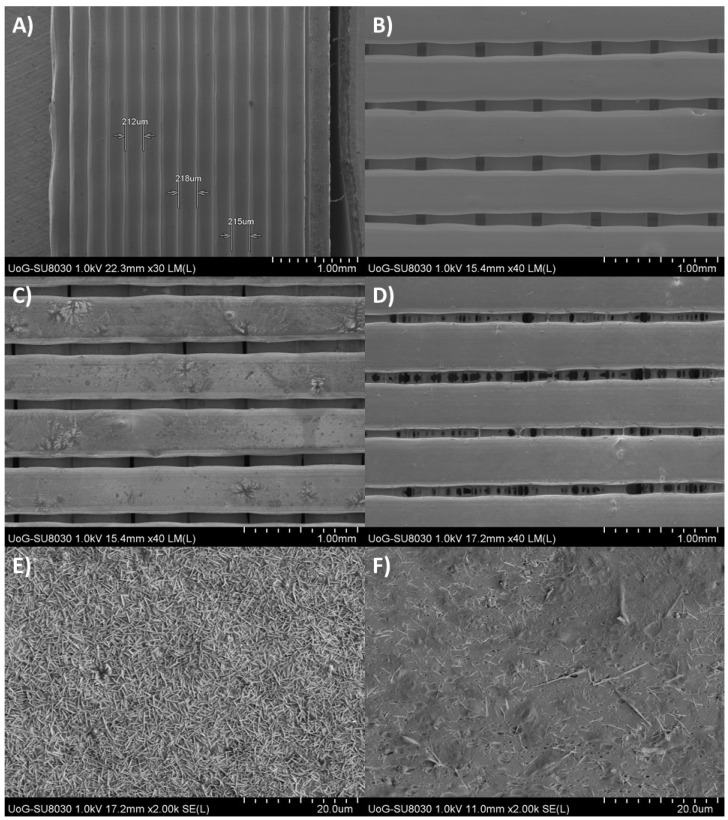
SEM images of (**A**) plain PVA tablet side view, (**B**) plain PVA surface, (**C**) P100 surface, (**D**) P70 surface (**E**) P100 crystals, and (**F**) P70 crystals.

Figure 7 illustrates SEM micrographs of the plain 3D printed tablets and inkjet printed tablets with P100, and P70 drug solutions. The layered structure of the plain tablets showed a consistent layer thickness varying from 212 to 218 μm (Figure 7A). In agreement with the infill pattern selected, the tablets present a surface with parallel and perpendicular lines (Figure 7B). This is because the lines in a layer are printed in a parallel manner, and the different layers are located perpendicularly on top of each other. As shown in Figure 7C,D, the deposition of both ethanolic (minoxidil) solutions has altered the surface of the tablets. The inkjet application of P100 showed large amounts of crystals on its surface (Figure 7C). In contrast P70, appeared with just a few crystals on the surface (Figure 7D). By increasing the magnification, it was possible to confirm the difference in the drug crystallization on the tablet surface as a result of P100 (Figure 7E) and P70 (Figure 7F) dispensed solutions.

The difference on the crystallized minoxidil on the tablet surface can only be explained by the dissolving capacity of P70 on the PVA polymer compared to P100. It is clear that P70 partially dissolve PVA and hence allowed the drug absorption on the tablet layers. This observation is in good agreement with the dissolution profiles obtained for both solvent grades.

## 4. Conclusions

The 3D FDM technology was coupled with inkjet printing to produce drug-loaded 3D printed tablets wherein FDM was used to fabricate the plain PVA tablet followed by the dispensing of minoxidil ethanolic solutions by the inkjet printer. The study showed that the physical state of the deposited drug is affected by the miscibility of the solvent with the polymer substrate. As a result, minoxidil was found to be in crystalline state on the tablet surface or absorbed in the polymer matrix resulting in different release profiles. The approach can be developed for personalized medicines by further exploring other drugs or combinations of drug/polymer inks for controlled release profiles.

## Figures and Tables

**Table 1:** Results of the printing reproducibility.

Parameter	Diameter (mm)	Height (mm)	Depth (mm)
Mean	12.00	3.00	2.01
SD	0.09	0.02	0.02
RSD	0.74	0.70	0.94

SD: standard deviation; RSD: relative standard deviation.

**Table 2:** Evaluation of the weight variation of the minoxidil tablets.

	P70		P100	
	Tablet Weight (mg)	Deviation %	Tablet Weight (mg)	Deviation %
Average	238.9	0.00	234.9	0.00
Median	236.3	−1.09	235.7	0.33
Maximum	247.5	3.60	242.9	3.41
Minimum	233.5	−2.26	228.2	−2.85
SD	4.92	2.06	4.63	1.97

P70: tablet produced with ethanol 70% *v/v*; P100: tablet produced with absolute ethanol; SD: standard deviation.

## Data Availability

Not applicable.
